# Supplementary material for: Pyridoxine induces glutathione synthesis via PKM2-mediated Nrf2 transactivation and confers neuroprotection
Source: Nat Commun. 2020 Feb 18;11:941. doi: 10.1038/s41467-020-14788-x (PMC7029000; doi:10.1038/s41467-020-14788-x)
Supplement: Supplementary file 1 — Supplementary Information [file 41467_2020_14788_MOESM1_ESM.pdf]

Supplementary Information

Pyridoxine induces glutathione synthesis via PKM2-mediated Nrf2 transactivation and confers neuroprotection

Yao Wei et al.

Table of contents

Supplementary figures 1-8

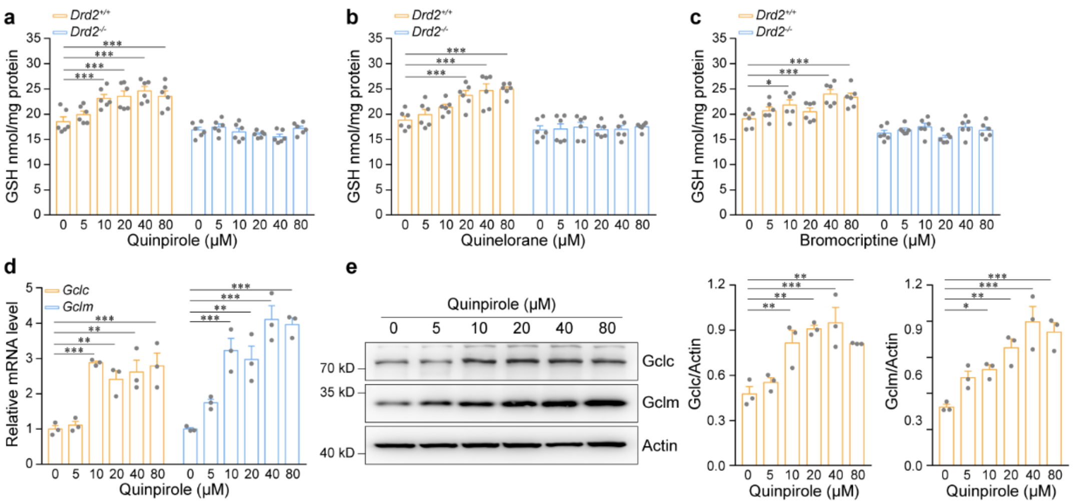

Supplementary Fig. 1 Astrocytic DRD2 facilitates Gclc/Gclm expression and GSH synthesis. **a-c** GSH levels in astrocytes from wild-type mice or *Drd2*-knockout mice after treatment with 0, 5, 10, 20, 40, or 80 µM quinpirole (a), quinelorane (b) or bromocriptine (c) for 12 h. Six independent experiments per condition. **d, e** Astrocytes were treated with 0, 5, 10, 20, 40, or 80 µM quinpirole, and *Gclc*/*Gclm* mRNA was analyzed by qRT-PCR (d) and immunoblotting with actin as a loading control (e). Three independent experiments per condition. Data are presented as the mean ± s.e.m. **P* < 0.05, ***P* < 0.01, ****P* < 0.001. One-way ANOVA with Dunnett's multiple comparisons test (d, e). Two-way ANOVA with Dunnett's multiple comparisons test (a-c). Blots are included in the source data file.

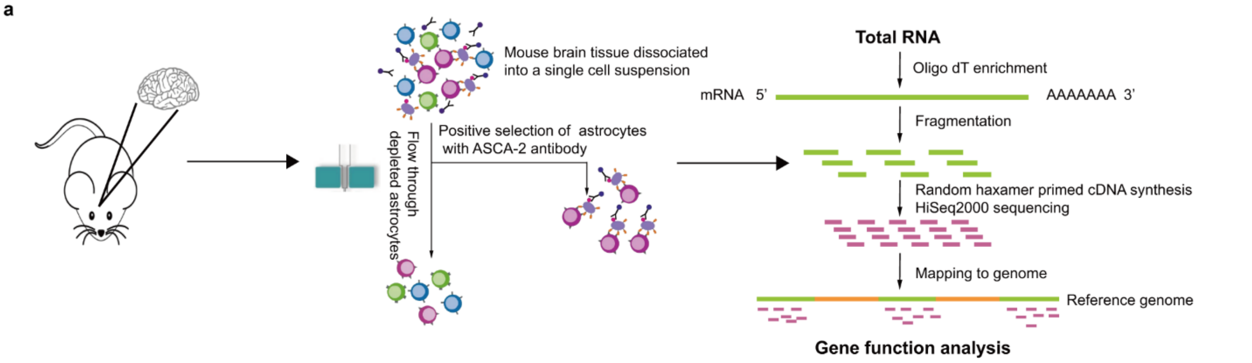

b

SampleID	ReadSum	BaseSum	GC (%)	N (%)	Q20 (%)	Q30 (%)
Saline1	30,061,484	8,975,610,830	53.24	0	98.18	94.96
Saline2	30,994,567	9,261,691,018	53.12	0	98.22	95.05
Saline3	28,628,382	8,547,435,360	53.26	0	98.11	94.79
Quinpirole1	29,486,695	8,809,333,482	53.09	0	98.13	94.84
Quinpirole2	28,315,561	8,458,148,366	53.24	0	98.11	94.79
Quinpirole3	24,840,891	7,411,921,558	53.34	0	98.05	94.66

c

SampleID	Total Reads	Mapped Reads	Uniq Mapped Reads	Multiple Mapped Reads
Saline1	60,122,968	57,603,068 (95.81%)	55,959,121(93.07%)	1,643,947 (2.73%)
Saline2	61,989,134	59,500,897 (95.99%)	57,678,981(93.05%)	1,821,916 (2.94%)
Saline3	57,256,764	54,798,719 (95.71%)	53,094,019(92.73%)	1,704,700 (2.98%)
Quinpirole1	58,973,390	56,335,602 (95.53%)	54,490,548(92.40%)	1,845,054 (3.13%)
Quinpirole2	56,631,122	54,066,515 (95.47%)	52,245,021(92.25%)	1,821,494 (3.22%)
Quinpirole3	49,681,782	47,306,492 (95.22%)	45,773,014(92.13%)	1,533,478 (3.09%)

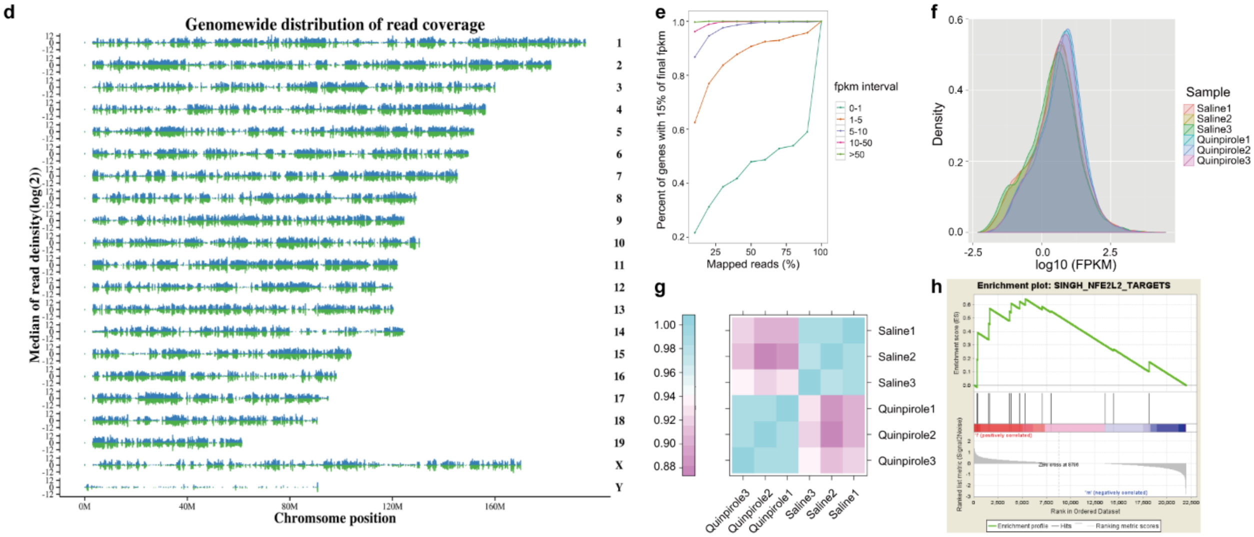

Supplementary Fig. 2 RNA-seq in astrocytes after DRD2 activation. **a** Schematic diagram of astrocyte separation from the adult mouse brain and RNA-seq. **b** Quality control of RNA-seq data. **c** The efficiency of clean reads mapped to the reference sequence. **d** Representative genome-wide distribution of read coverage. **e** Representative saturation analysis of RNA-seq data. **f** Density distribution of FPKM. **g** Pearson's correlation coefficient of RNA-seq data. **h** Gene set enrichment analysis plot of Nrf2 targets.

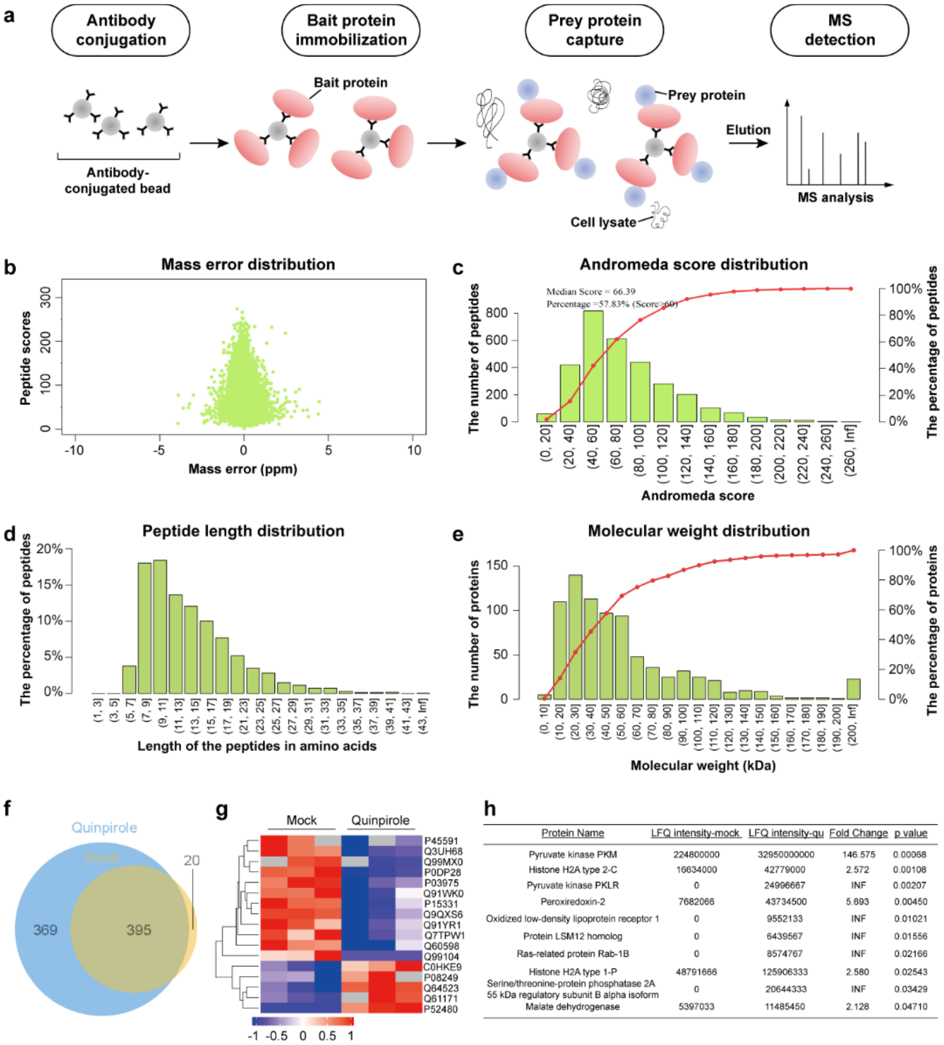

Protein Name	LFQ intensity-mock	LFQ intensity-qu	Fold Change	p value
Pyruvate kinase PKM	224800000	32950000000	146.575	0.00068
Histone H2A type 2-C	16634000	42779000	2.572	0.00108
Pyruvate kinase PKLR	0	24996667	INF	0.00207
Peroxiredoxin-2	7682066	43734500	5.693	0.00450
Oxidized low-density lipoprotein receptor 1	0	9552133	INF	0.01021
Protein LSM12 homolog	0	6439567	INF	0.01556
Ras-related protein Rab-1B	0	8574767	INF	0.02166
Histone H2A type 1-P	48791666	125906333	2.580	0.02543
Serine/threonine-protein phosphatase 2A 55 kDa regulatory subunit B alpha isoform	0	20644333	INF	0.03429
Malate dehydrogenase	5397033	11485450	2.128	0.04710

Supplementary Fig. 3 Mass spectrometry quantification of proteins that bind to Nrf2 after DRD2 activation. **a** Schematic diagram of the anti-Nrf2 pull-down assay and label-free detection. **b** Mass error distribution of peptide scores. **c** Andromeda score distribution of the identified peptides. **d** Peptide length distribution. **e** The molecular weight distribution of the identified proteins. **f** Venn diagram showing the overlapping proteins identified under two conditions. **g** Hierarchical clustering of the differentially expressed protein profiles. **h** Proteins that bound Nrf2 with a more than 2-fold greater abundance and a p-value less than 0.05 after DRD2 activation.

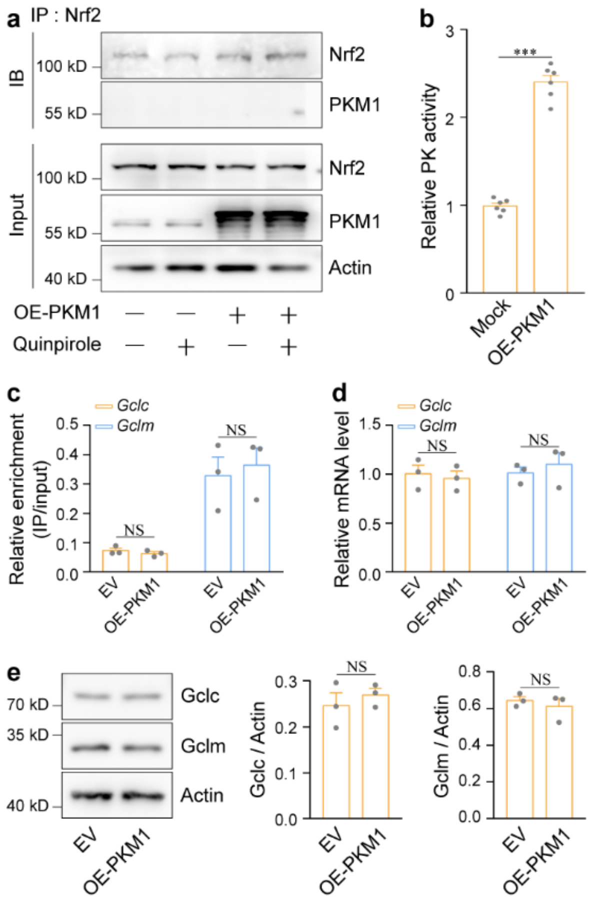

Supplementary Fig. 4 PKM1 does not bind Nrf2 or affect its transcriptional activity. **a-e** Primary astrocytes transfected with adenovirus overexpressing empty vector (EV) or PKM1 (OE-PKM1) were stimulated with 10 µM quinpirole for 12 h. Immunoblot analysis of PKM1 in cell lysates immunoprecipitated with a Nrf2 antibody (a). Pyruvate kinase activity (b). The amount of anti-Nrf2-immunoprecipitated DNA was analyzed by qRT-PCR with primers flanking the *Gclc* and *Gclm* promoter regions (c). *Gclc* and *Gclm* mRNA detected by qRT-PCR (d). Immunoblotting with actin as a loading control (left) and densitometric analysis of Gclc/Gclm (right) (e). Immunoblotting and qRT-PCR: three independent experiments; pyruvate kinase activity assay: six independent experiments. Data are presented as the mean ± s.e.m. ***$P < 0.001$, NS, not significant. Student's two-tailed unpaired *t*-test (b-e).

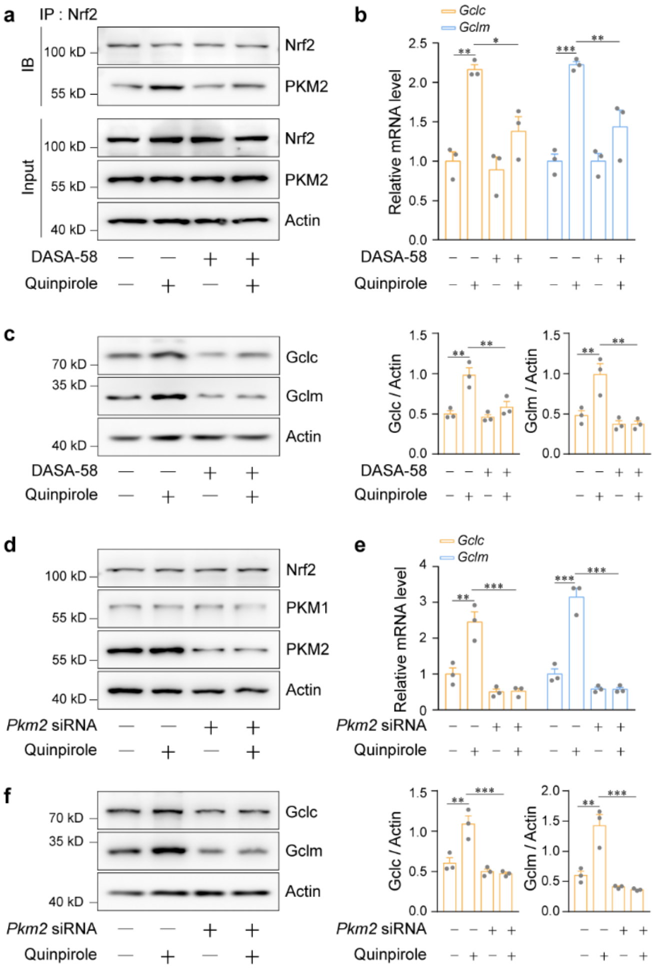

Supplementary Fig. 5 DRD2 activation promotes Nrf2 transactivation via PKM2. **a-c** Primary astrocytes were pretreated with 50 μM DASA-58 for 1 h and then stimulated with 10 μM quinpirole for 12 h. Immunoblot analysis of PKM2 in cell lysates immunoprecipitated with an Nrf2 antibody (a). *Gclc* and *Gclm* mRNA detected by qRT-PCR (b). Immunoblotting with actin as a loading control (left) and densitometric analysis of Gclc/Gclm (right) (c). Immunoblotting and qRT-PCR: three independent experiments. **d-f** Primary astrocytes transfected with *Pkm2*-specific siRNA were stimulated with 10 μM quinpirole for 12 h. Immunoblotting with actin as a loading control (d). *Gclc* and *Gclm* mRNA detected by qRT-PCR (e). Immunoblotting with actin as a loading control (left) and densitometric analysis of Gclc/Gclm (right) (f). Immunoblotting and

qRT-PCR: three independent experiments. Data are presented as the mean ± s.e.m. *$P < 0.05$, **$P < 0.01$, ***$P < 0.001$. One-way ANOVA with Tukey's multiple comparisons test (b, c, e and f).

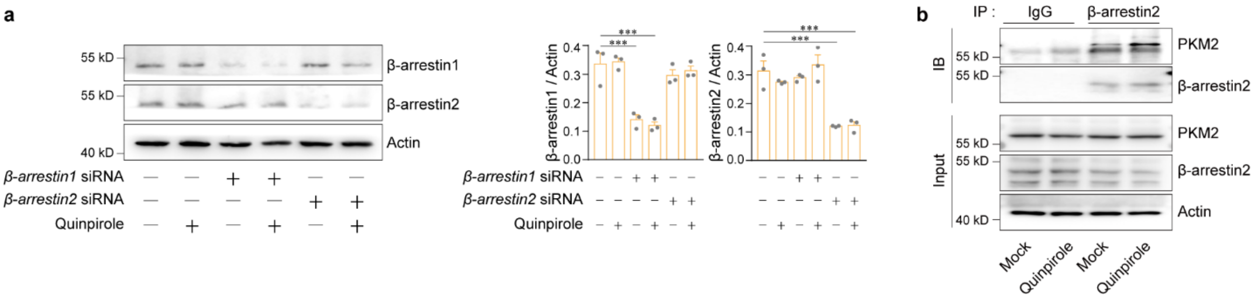

Supplementary Fig. 6 DRD2 activation triggers β-arrestin2 to bind and dimerize PKM2. **a** Primary astrocytes transfected with *β-arrestin1*- or *β-arrestin2*-specific siRNA were stimulated with 10 μM quinpirole for 12 h. Immunoblotting with actin as a loading control (left) and densitometric analysis of β-arrestin1/β-arrestin2 (right). Three independent experiments per condition. **b** Immunoblot analysis of PKM2 in 10 μM quinpirole-stimulated astrocyte lysates immunoprecipitated with the β-arrestin2 antibody. Three independent experiments per condition. Data are presented as the mean ± s.e.m. ***$P < 0.001$. One-way ANOVA with Dunnett's multiple comparisons test (a).

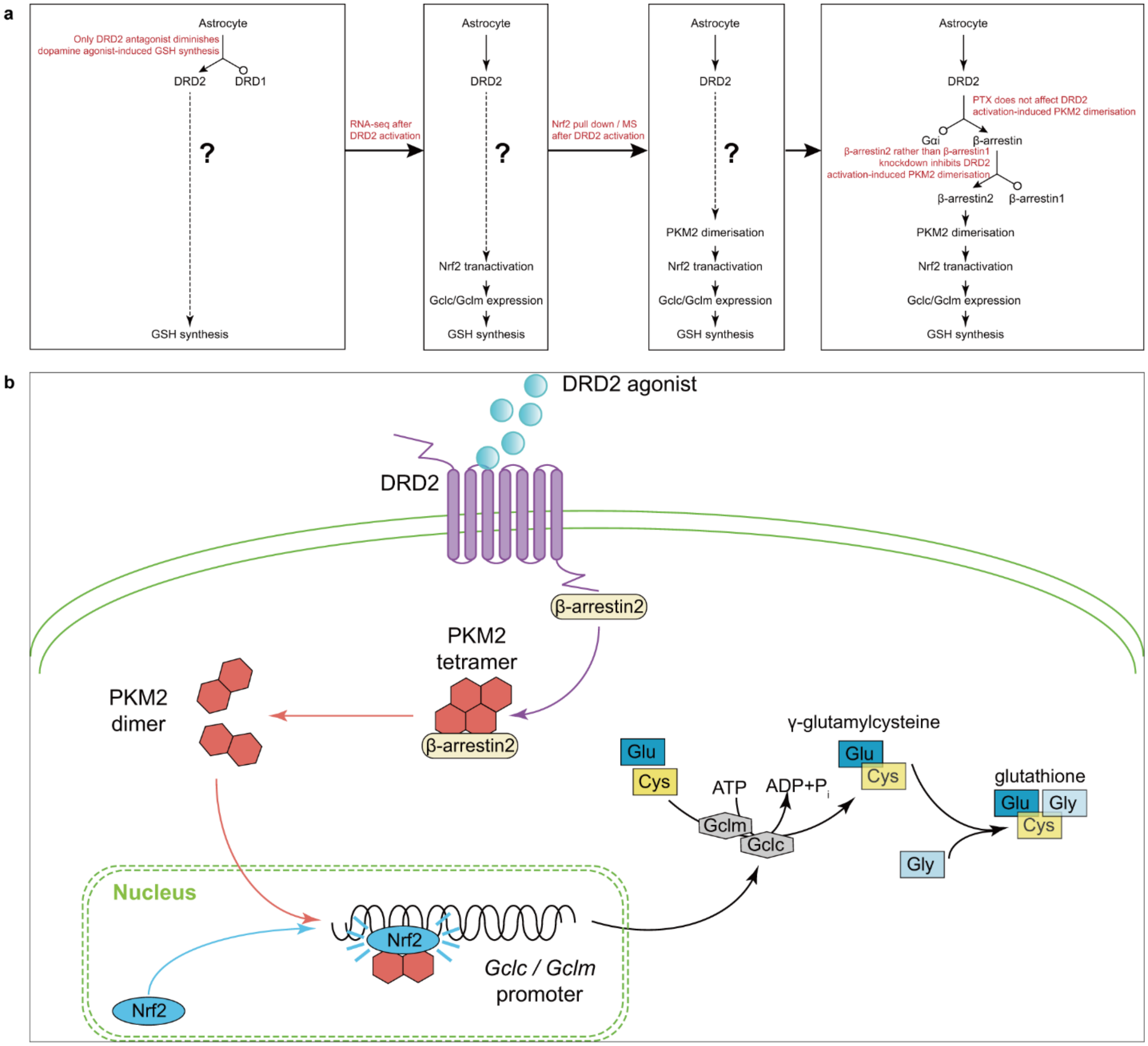

Supplementary Fig. 7 Schematic summary**. a** Schematic description of the experimental procedure. **b** Schematic summarizing the mechanism by which DRD2 activation triggers the binding of β-arrestin2 to PKM2 and promotes PKM2 dimerization, resulting in Nrf2 transactivation and GSH biosynthesis.

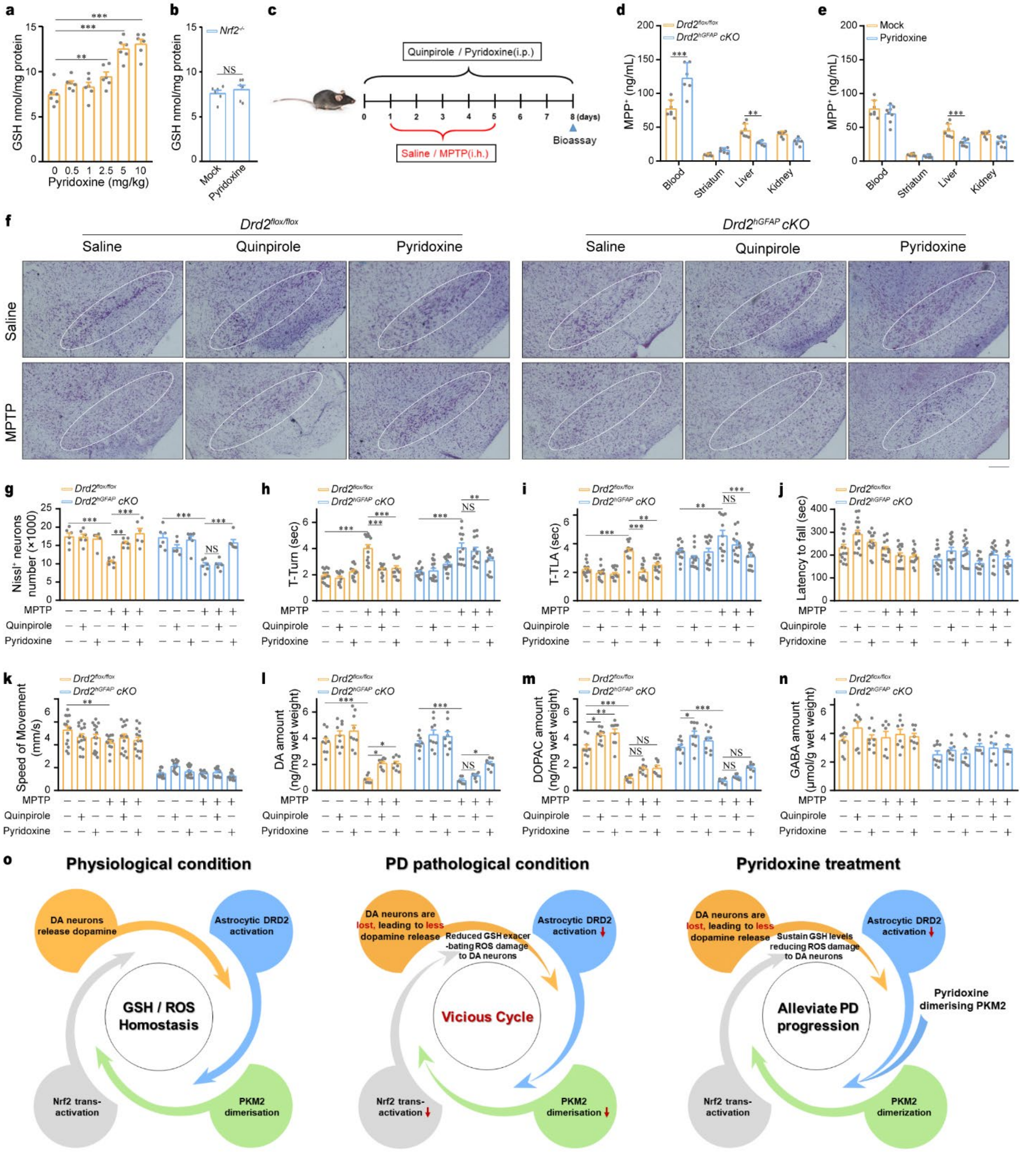

Supplementary Fig. 8 Pyridoxine reduces dopaminergic neuron loss in the PD mouse model. **a** Striatal GSH levels in wild-type mice administered 0.5, 1, 2.5, 5, or 10 mg kg^{-1} pyridoxine for 7 days. n=6 mice per group. **b** Striatal GSH levels in *Nrf2*-knockout mice administered 5 mg kg^{-1} pyridoxine for 7 days. n=6 mice per group. **c** Experimental procedure and different drug

administration schemes. **d-n** Drd2$^{flox/flox}$ and Drd2^{hGFAP} *cKO* mice were used to generate an MPTP-induced PD mouse model (20 mg kg^{-1} i.h., 5 d), which was treated with continuous infusion of quinpirole (5 mg kg^{-1} i.p.) or pyridoxine (5 mg kg^{-1} i.p.). MPP^{+} levels in the blood, striatum, liver, and kidney detected by HPLC (d, e). Nissl staining of neurons (f). Quantification of Nissl-positive neurons (g). Times required for mice to turn around (h) and descend a pole (i) in the pole test. Latency time in the rotarod test (j). Mouse activities in an open field within 5 min (k). Striatal dopamine (l), DOPAC (m) and GABA (n) levels detected by HPLC. n=6-8 mice per group for HPLC; n=6 mice per group for Nissl staining; n=12-15 mice per group for behavioral assays. Scale bar, 200 µm. **o** Schematic summarizing the vicious cycle of decreased dopamine release—DRD2 signaling deficiency—decreased GSH synthesis—oxidative stress—dopaminergic neuron death in PD and showing how directly dimerizing PKM2 is a possible strategy for breaking this vicious cycle and potentially alleviating PD progression. Data are presented as the mean ± s.e.m. *$P < 0.05$, **$P < 0.01$, ***$P < 0.001$, NS, not significant. One-way ANOVA with Dunnett's multiple comparisons test (a). Two-way ANOVA with Sidak's multiple comparisons test (d, e) or with Tukey's multiple comparisons test (g-n). Student’s two-tailed unpaired *t*-test (b).
