# Supplementary material for: Pyridoxine induces glutathione synthesis via PKM2-mediated Nrf2 transactivation and confers neuroprotection
Source: Nat Commun. 2020 Feb 18;11:941. doi: 10.1038/s41467-020-14788-x (PMC7029000; doi:10.1038/s41467-020-14788-x)
Supplement: Supplementary file 3 — Description of Additional Supplementary Files [file 41467_2020_14788_MOESM3_ESM.pdf]

Description of Additional Supplementary Files

File name: Supplementary Data 1
Description: RNA sequence data referring to Fig. 1g and Supplementary Fig. 2.

File name: Supplementary Data 2
Description: Label-free mass spectrometry data referring to Fig. 2a and Supplementary Fig. 3.

File name: Supplementary Data 3
Description: NanoDSF data referring to Fig. 5a.
