# Supplementary material for: Pyridoxine induces glutathione synthesis via PKM2-mediated Nrf2 transactivation and confers neuroprotection
Source: Nat Commun. 2020 Feb 18;11:941. doi: 10.1038/s41467-020-14788-x (PMC7029000; doi:10.1038/s41467-020-14788-x)
Supplement: Supplementary file 7 — Reporting Summary [file 41467_2020_14788_MOESM7_ESM.pdf]

nature research

Corresponding author(s): Gang Hu

Last updated by author(s): 2020-1-2

Reporting Summary

Nature Research wishes to improve the reproducibility of the work that we publish. This form provides structure for consistency and transparency in reporting. For further information on Nature Research policies, see Authors & Referees and the Editorial Policy Checklist.

Statistics

For all statistical analyses, confirm that the following items are present in the figure legend, table legend, main text, or Methods section.

n/a	Confirmed	
☐	☒	The exact sample size (*n*) for each experimental group/condition, given as a discrete number and unit of measurement
☐	☒	A statement on whether measurements were taken from distinct samples or whether the same sample was measured repeatedly
☐	☒	The statistical test(s) used AND whether they are one- or two-sided<br>*Only common tests should be described solely by name; describe more complex techniques in the Methods section.*
☒	☐	A description of all covariates tested
☐	☒	A description of any assumptions or corrections, such as tests of normality and adjustment for multiple comparisons
☐	☒	A full description of the statistical parameters including central tendency (e.g. means) or other basic estimates (e.g. regression coefficient) AND variation (e.g. standard deviation) or associated estimates of uncertainty (e.g. confidence intervals)
☐	☒	For null hypothesis testing, the test statistic (e.g. *F*, *t*, *r*) with confidence intervals, effect sizes, degrees of freedom and *P* value noted<br>*Give P values as exact values whenever suitable.*
☒	☐	For Bayesian analysis, information on the choice of priors and Markov chain Monte Carlo settings
☒	☐	For hierarchical and complex designs, identification of the appropriate level for tests and full reporting of outcomes
☒	☐	Estimates of effect sizes (e.g. Cohen's *d*, Pearson's *r*), indicating how they were calculated

Our web collection on statistics for biologists contains articles on many of the points above.

Software and code

Policy information about availability of computer code

Data collection	Data were collected on Applied Biosystems StepOnePlus PCR system using StepOne software version 2.3 (qRT-PCR), on Beckman Gallios using Kaluza version 1.3 (flow cytometry), on confocal laser scanning microscopy platform Leica TCS SP8 using AF6000 modular system (immunofluorescence), and using ImageQuant LAS 4000 mini version 1.3 (immunoblotting).
Data analysis	Data analyses were performed using GraphPad PRISM 7 software (statistical analysis), ImageJ (densitometric analysis), MaxQuant software version 1.3.0.5 (label-free), MBF Bioscience Stereo Investigator version 2018 (cell counting) and PR.ThermControl version 2.1.5 (NanoDSF).

For manuscripts utilizing custom algorithms or software that are central to the research but not yet described in published literature, software must be made available to editors/reviewers. We strongly encourage code deposition in a community repository (e.g. GitHub). See the Nature Research guidelines for submitting code & software for further information.

Data

Policy information about availability of data

All manuscripts must include a data availability statement. This statement should provide the following information, where applicable:

- Accession codes, unique identifiers, or web links for publicly available datasets
- A list of figures that have associated raw data
- A description of any restrictions on data availability

The original and analysed datasets generated during the current study, and the codes used to analyse them, are available from the corresponding author upon reasonable request. There is no restriction on material availability. We include a statement on data availability in our manuscript, as required by Nature policy.

Field-specific reporting

Please select the one below that is the best fit for your research. If you are not sure, read the appropriate sections before making your selection.

☒ Life sciences ☐ Behavioural & social sciences ☐ Ecological, evolutionary & environmental sciences

For a reference copy of the document with all sections, see nature.com/documents/nr-reporting-summary-flat.pdf

Life sciences study design

All studies must disclose on these points even when the disclosure is negative.

Sample size	No statistical methods were used to predetermine the sample size. Required sample sizes were estimated based on our experience performing similar experiments in previous publications.
Data exclusions	No data were excluded.
Replication	Consistent results obtained from at least three biological replicates with more than two technical replicates per experiment were used in the manuscript.
Randomization	Animals were randomly assigned to either experimental or control groups.
Blinding	Investigators were blinded to the group allocation and treatment for animal experiments when counting cells in immunohistochemistry analysis.

Reporting for specific materials, systems and methods

We require information from authors about some types of materials, experimental systems and methods used in many studies. Here, indicate whether each material, system or method listed is relevant to your study. If you are not sure if a list item applies to your research, read the appropriate section before selecting a response.

Materials & experimental systems

n/a	Involved in the study	
☐	☒	Antibodies
☒	☐	Eukaryotic cell lines
☒	☐	Palaeontology
☐	☒	Animals and other organisms
☒	☐	Human research participants
☒	☐	Clinical data

Methods

n/a	Involved in the study	
☒	☐	ChIP-seq
☐	☒	Flow cytometry
☒	☐	MRI-based neuroimaging

Antibodies

Antibodies used	Antibodies used for immunoblotting studies include: rabbit anti-PKM1 monoclonal (1:1000, Cell Signaling Technology, 7067), rabbit anti-PKM2 monoclonal (1:1000, Cell Signaling Technology, 4053), rabbit anti-Nrf2 monoclonal (1:1000, Cell Signaling Technology, 12721), rabbit anti-β-arrestin1 monoclonal (1:1000, Cell Signaling Technology, 12697), rabbit anti-β-arrestin2 monoclonal (1:1000, Cell Signaling Technology, 3857), rabbit anti-β3-tubulin monoclonal (1:1000, Cell Signaling Technology, 4466), rabbit anti-β-actin monoclonal (1:1000, Cell Signaling Technology, 4970), rabbit anti-Gclc polyclonal (1:1000, Proteintech, 12601-1-AP), rabbit anti-Gclm polyclonal (1:1000, Proteintech, 14241-1-AP), and mouse anti-GFAP monoclonal (1:1000, Millipore, MAB360).<br>Antibodies used for co-IP studies include: mouse anti-Nrf2 monoclonal (1:1000, Proteintech, 66504-1-lg) and mouse anti-β-arrestin2 monoclonal (1:500, Santa Cruz, sc-514791).<br>Antibodies used for Proximity ligation assay include: mouse anti-Nrf2 monoclonal (1:50, Proteintech, 66504-1-lg) and rabbit anti-PKM2 monoclonal (1:50, Cell Signaling Technology, 4053).<br>Antibodies used for immunohistochemistry or immunofluorescence include: mouse anti-TH-2 monoclonal (1:1000, Sigma Aldrich, T1299), mouse anti-GFAP monoclonal (1:500, Millipore, MAB360), mouse anti-NeuN monoclonal (1:500, Millipore, MAB377), rabbit anti-PKM1 monoclonal (1:1000, Cell Signaling Technology, 7067), rabbit anti-PKM2 monoclonal (1:50, Cell Signaling Technology, 4053).
Validation	All antibodies above are in everyday use or have been validated in other literature for use in mice. We also confirmed that each antibody for immunofluorescence stained in a typical cellular pattern and brain-wide distributions at its target proteins. We provide detailed catalogue number, source, and dilution for each antibody used in the current study.

Animals and other organisms

Policy information about studies involving animals; ARRIVE guidelines recommended for reporting animal research

Laboratory animals	Adult or neonatal C57BL/6 mice were purchased from the Model Animal Research Center of Nanjing University. Drd2-floxed mice were created by Shanghai Research Center for Model Organisms. Drd2-knockout mice (Jax Stock No: 003190) and hGFAP-Cre transgenic mice on a C57BL/6 genetic background, which were initially derived from GFAP-Cre mice (Jax Stock No: 004600), were a gift from Jiawei Zhou. Nrf2-knockout mice (Jax Stock No: 017009) were a gift from Peng Cao. β-Arrestin2-knockout mice (Jax Stock No: 011130) were obtained from Gang Pei. Three-month-old male Drd2flox/flox and Drd2hGFAP cKO mice were used to generate a subacute MPTP-induced PD model. All animal care and procedures were performed following national and international guidelines and were approved by the Animal Resource Centre, Nanjing Medical University.
Wild animals	No wild animals used.
Field-collected samples	No field-collected samples used.
Ethics oversight	No ethics oversight.

Note that full information on the approval of the study protocol must also be provided in the manuscript.

Flow Cytometry

Plots

Confirm that:

- ☒ The axis labels state the marker and fluorochrome used (e.g. CD4-FITC).
- ☒ The axis scales are clearly visible. Include numbers along axes only for bottom left plot of group (a 'group' is an analysis of identical markers).
- ☒ All plots are contour plots with outliers or pseudocolor plots.
- ☒ A numerical value for number of cells or percentage (with statistics) is provided.

Methodology

Sample preparation	A citation describing sample preparation in included in the Methods section.
Instrument	Beckman Gallios.
Software	Beckman Kaluza version 1.3.
Cell population abundance	No sorting was conducted.
Gating strategy	FSC/SSC was used to discern single cells from doublets/multiple cells. Samples without fluorescent staining were used to establish boundaries between negative and positive cells. No sequential gating was performed.

- ☐ Tick this box to confirm that a figure exemplifying the gating strategy is provided in the Supplementary Information.
